# Evaluation of the importance of mixing during preparation of antibiotic infusions

**DOI:** 10.1186/s40360-022-00562-w

**Published:** 2022-04-08

**Authors:** Ina Barzel, Janique Gabriëlle Jessurun, Soma Bahmany, Paul Hugo Marie van der Kuy, Birgit Catharina Peter Koch, Nicole Geertruida Maria Hunfeld

**Affiliations:** 1 Department of Hospital Pharmacy, Erasmus MC, University Medical Center Rotterdam, P.O. Box 2040, 3000 CA Rotterdam, the Netherlands; 2 Department of Intensive Care, Erasmus MC, University Medical Center Rotterdam, Rotterdam, the Netherlands

**Keywords:** Cefuroxime, Flucloxacillin, Meropenem, Vancomycin, Antibiotics, Mixing, Infusion, Concentration, Admixture, Intravenous admixture

## Abstract

**Background:**

The mixing step after medication addition to the infusion bag is frequently omitted during the preparation of drug infusions. However, the importance of mixing when preparing antibiotic infusions is still unknown.

**Methods:**

The primary aim of this study was to assess the importance of the mixing step by comparing the concentrations of unmixed antibiotic infusions (cefuroxime, flucloxacillin, meropenem, and vancomycin) with the declared concentration at regular intervals during infusion. The secondary aim was to compare concentrations between preparation sites (hospital pharmacy versus clinical ward). Infusion bags were run through electronic infusion pumps. For cefuroxime, flucloxacillin, and meropenem, samples were collected 1, 15, and 20 min after starting the administration (infusion duration: 30 min). For vancomycin, samples were collected after 1, 60, and 110 min (infusion duration: 120 min). Vancomycin concentrations were measured using the Architect c4000 analyser and other concentrations using a validated UPC^2^-MS–MS multimethod.

**Results:**

The median concentrations of the four antibiotics were comparable to the declared concentration at all three time points. No significant differences were found between preparation sites.

**Conclusions:**

Spontaneous mixing occurred in the examined antibiotic solutions during normal handling.

## Background

The preparation of drug infusions is a complex process with multiple steps and therefore constitutes a risk for various errors. One error type is incomplete mixing after a drug solution is added to the infusion fluid. Studies on the prevalence of this error type are scarce and have shown inconsistent results, with error rates varying from 0%-79% [1–3].

However, there is no consensus in the literature on the importance of the mixing step after medication addition to the infusion bag. While many pharmacists consider the extent of mixing through diffusion and normal handling sufficient, several studies show that incomplete mixing can put patients at risk [4–7]. In a study of Thompson et al., the concentration of potassium chloride released at the start of administration was four times higher than the declared concentration (i.e. the concentration on the product label), while Deardorff et al. even stated a 30 times higher concentration at the beginning of infusion [4, 6].

Unfortunately, studies on the influence of mixing on frequently used antibiotics, such as cefuroxime and meropenem, are scarce. To our knowledge, only one study has been performed with antibiotics, i.e. with meropenem in a concentration of 1000 mg in 250 mL. In the meropenem study, slight spontaneous mixing occurred when mixing was omitted during preparation [8] .

Antibiotics differ in their physiochemical properties and therefore their potential to induce infusion-related reactions [9]. For several antibiotics, infusion of a concentration higher than the recommended concentration (e.g. due to high osmolarity or non-physiologic pH) may lead to an increased risk of pain at the injection site, irritation, or phlebitis [9, 10]. Another example of a possible adverse drug event that may occur is that rapid administration of vancomycin may lead to a red man syndrome, with symptoms such as flushing, rash, fever, dyspnoea, and cardiovascular shock [11]. In addition, with varying concentrations in an infusion bag, patients may receive an unknown dose if the infusion line is not flushed after administration, which is frequently the case in clinical practice [12, 13]. Hence, there is a need to investigate the importance of the mixing step on drug concentrations during infusion, especially for frequently prepared antibiotic infusions.

### Aim of the study

The aim of this experimental study was to examine the importance of the mixing step after medication addition to the infusion bag by comparing the concentrations of four unmixed antibiotic infusions (cefuroxime, flucloxacillin, meropenem, and vancomycin) at regular intervals during infusion with the declared concentration and between preparation sites (hospital pharmacy versus clinical ward).

## Methods

We conducted an experimental study in July 2020 in Erasmus MC, University Medical Center Rotterdam in Rotterdam, the Netherlands. This study was performed in the hospital pharmacy and a clinical ward unit in a simulation setting under the standard conditions of these environments. Ethical approval was not sought for the present study, because it does not involve human or animal subjects.

### Preparation of antibiotic infusions

The following frequently prepared antibiotics were examined: cefuroxime, flucloxacillin, meropenem, and vancomycin. The specifications of the products used are shown in Table 1.

**Table 1:** Used products of the examined antibiotic infusions

**Generic name**	**Commercial name**	**Dose per injection vial**	**Volume after reconstitution**	**Infusion fluid**^c^	**Declared concentration**
Cefuroxime	Cefuroxim^a^	1500 mg	16 mL	Normal saline 50 mL	1500 mg/66 mL (22.7 mg mL^−1^)
Flucloxacillin	Floxapen®^b^	1000 mg	20 mL	Normal saline 50 mL	1000 mg/70 mL (14.3 mg mL^−1^)
Meropenem	Meropenem^a^	1000 mg	20 mL	Normal saline 50 mL	1000 mg/70 mL (14.3 mg mL^−1^)
Vancomycin	Vancomycine^b^	1000 mg	20 mL	Normal saline 250 mL	1000 mg/270 mL (3.7 mg mL^−1^)

^a^ Manufacturer: Fresenius Kabi; Bad Homburg, Germany

^b^ Manufacturer: Aurobindo; Hyderabad, India

^c^ Manufacturer: Baxter International Inc.; Deerfield, Illinois, USA

Three pharmacy technicians prepared all four antibiotic infusions twice in each environment. Intravenous infusions were prepared in the laminar air flow (LAF) cabinets in the cleanrooms of the hospital pharmacy and on the standard workbenches in the medication room of the clinical ward. These two environments were examined, because they are conventional preparation areas in hospitals, while their transportation route and normal handling procedures differ. The pharmacy technicians were instructed to leave out the mixing step after adding the antibiotic to the infusion fluid. In the hospital pharmacy environment, the required drug volume was weighed so that a pharmacist (I.B.) could verify the right amount of the antibiotic. Until transport to the clinical ward, cefuroxime and meropenem infusions were stored between 2–8 °C and flucloxacillin and vancomycin at room temperature. In the clinical ward environment, a pharmacist (I.B.) checked the drug volume before it was added to the infusion fluid. The antibiotic infusions were stored at room temperature for half an hour before used for administration simulation. The following materials were used for preparation: needles (19 G × 1 ½’’, 1.1 mm × 40 mm, BD), syringes (20 mL, BD) and normal saline infusion bags (Viaflo 50 mL and 250 mL, Baxter International Inc.).

### Sample collection

The antibiotic infusions prepared in the hospital pharmacy were transported with the regular logistic chain to the clinical ward. The simulation of administration took place in an unoccupied patient room. Electronic infusion pumps (Infusomat® Space infusion pumps from B. Braun Medical; Melsungen, Germany) were used to reflect general clinical practice of intravenous drug administration. Before starting the administration, the infusion line was filled with antibiotic infusion solution. To mimic real-life conditions, the most conventional infusion time was set according to local protocols (based on the literature). In compliance with these protocols, cefuroxime, flucloxacillin, and meropenem should be administered in 15–30 min, while vancomycin > 500 mg should be administered in at least 2 h. For cefuroxime, flucloxacillin, and meropenem, the total infusion duration was set at 30 min and samples were collected at three different time points during infusion (1 min, 15 min, and 20 min after starting the administration). For vancomycin, the infusion time was set at 120 min and samples were collected after 1 min, 60 min, and 110 min. Sample collection took place at only three different time points (at the beginning, middle, and end of the infusion) to minimise handling, as handling could affect the degree of mixing. We considered three different points sufficient to show any variations in drug concentration if present. The first two samples were collected from the infusion line. To prevent the infusion pump from stopping to function at the end of the infusion, the third sample was taken directly from the infusion bag. The infusion pump generated an alarm if all of the remaining solution was present in the infusion line, which prevented further suction of solution from the infusion bag and subsequently solution delivery from the infusion line. All samples were stored at -80 °C until analysis (Innova U535 ULT Freezer from New Brunswick Scientific™; Edison, USA).

### Concentration measurements

Samples were diluted in MilliQ water to obtain concentrations in the validated range. For the determination of vancomycin, samples were measured on the Architect c4000 analyser (Abbott Laboratories; Abbott Park, Illinois, USA). A selective, sensitive, and robust ultra-performance convergence chromatography tandem mass spectrometry (UPC^2^-MS–MS) multimethod was used for the determination of flucloxacillin, meropenem, and cefuroxime. Compounds were first separated based on their polarity using super critical fluid chromatography (SFC). After this stage, compounds are separated by their m/z ratios in the mass spectrometer. The SFC was a Waters Acquity UPC^2^ system (Waters Corporation; Milford, Massachusetts, USA) that was coupled to a Xevo TQ-S micro system (Waters Corporation; Milford, Massachusetts, USA). This method is validated for the analysis of blood samples according to the US Food and Drug Administration guidelines in the ISO15189 accredited laboratory of Erasmus MC. A summary of the quantification limits of all compounds is shown in Table 2.

**Table 2:** Quantification limits of the compounds for concentration measurements

Compound	Lower Limit of Quantitation (LLOQ) (mg L^−1^)	Upper Limit of Quantitation (ULOQ) (mg L^−1^)
Cefuroxime	1.25	50.0
Flucloxacillin	1.0	123.0
Meropenem	1.6	65.0
Vancomycin	0.7	400.0

### Outcomes

The primary outcomes were the median concentrations of each type of antibiotic infusion measured at the first, second, and third time point during administration compared to the declared concentration. The secondary outcomes were the median concentrations of each type of antibiotic at the three time points compared between preparation sites (hospital pharmacy versus clinical ward).

### Data analysis

Descriptive statistics were used to compare the measured concentrations at the three time points of infusion with the declared concentration. The Mann–Whitney *U* test was used to compare the concentrations at the three time points for each type of antibiotic infusion by preparation site. For all statistical analyses, a significance level of 0.05 was set. Data were analysed using IBM SPSS Statistics®, software version 25 (IBM Corporation; Armonk, New York, USA).

## Results

The median sample concentrations of the four antibiotics cefuroxime, flucloxacillin, meropenem, and vancomycin at all three infusion time points stratified by preparation site are shown in Table 3.

**Table 3:** Concentrations of antibiotic infusions during administration; infusions prepared in both a hospital pharmacy and clinical ward without mixing the final solution

Characteristics			Sample collection time	Hospital pharmacy		Clinical ward		Hospital pharmacy versus clinical ward
*Infusion type*^a^	*Declared concentration*	*Infusion duration*	*Sample collection time after start of the administration*	*n*^b^	*Sample concentration, median (IQR)* *in mg mL*^*−1*^	*n*^b^	*Sample concentration, median (IQR)* *in mg mL*^*−1*^	*p value*^c^
**Cefuroxime** *1500 mg, 66 mL*	22.7 mg mL^−1^	30 min	1 min	6	19.5 (13.2–27.9)	6	23.8 (23.4–24.4)	.240
			15 min		21.2 (14.0–30.2)		23.5 (21.2–25.3)	.937
			20 min		15.7 (15.0–21.2)		22.3 (19.8–25.1)	.093
**Flucloxacillin** *1000 mg, 70 mL*	14.3 mg mL^−1^	30 min	1 min	6	15.9 (14.6–17.0)	6	16.3 (14.9–17.2)	.818
			15 min		15.7 (14.9–16.6)		16.3 (15.4–18.2)	.485
			20 min		16.1 (14.7–16.9)		16.2 (14.3–17.2)	.818
**Meropenem** *1000 mg, 70 mL*	14.3 mg mL^−1^	30 min	1 min	6	13.3 (12.6–13.6)	6	13.5 (11.5–13.7)	.818
			15 min		12.9 (12.3–13.2)		13.4 (12.1–13.6)	.310
			20 min		12.1 (11.5–13.1)		13.8 (12.0–13.9)	.240
**Vancomycin** *1000 mg, 270 mL*	3.7 mg mL^−1^	120 min	1 min	6	3.2 (3.0–3.3)	6	3.3 (3.2–3.6)	.394
			60 min		3.3 (3.2–3.4)		3.3 (3.2–3.6)	.485
			110 min		3.0 (2.8–3.2)		3.2 (3.1–3.6)	.180

*IQR* Interquartile range

^a^ Normal saline infusion bags of 50 mL (cefuroxime, flucloxacillin, meropenem) and 250 mL (vancomycin) were used

^b^ All four antibiotics were produced twice in each environment by three different pharmacy technicians

^c^ The Mann–Whitney *U* test was used to estimate *p* values

All median concentrations of the four antibiotics were comparable to the declared concentration at the beginning, middle, and end of administration. Deviations were less than 20% from the declared concentration, except for the median concentration of cefuroxime at the end of infusion, which deviated 30.8% from the declared concentration. No statistically significant differences in drug concentration were found between preparation sites at all three time points.

## Discussion

For each of the four investigated antibiotics (cefuroxime, flucloxacillin, meropenem, and vancomycin) prepared in the hospital pharmacy and clinical ward, median concentrations were comparable to the declared concentration at the beginning, middle, and end of infusion, which suggests spontaneous mixing to occur. Also, no significant differences were found between preparation sites.

Previous studies in different drugs on the importance of the mixing step after medication addition to the infusion bag show conflicting results [4–7, 14–16]. Many studies, mainly performed with concentrated electrolytes, show that omitting this step leads to inhomogeneous infusions and usually high concentrations in the first period of discharge, which subsequently may lead to patient harm, e.g. cardiac arrest after fast administration of potassium chloride [4–7, 14, 15]. Another issue that could arise with varying drug concentrations during infusion is that patients may receive an incomplete and unknown dose when flushing after administration is omitted [12, 13]. The risks with antibiotics may differ from concentrated electrolytes, but infusion of a high concentration of antibiotic solution has the potential to lead to infusion-related reactions, such as pain at the injection site and phlebitis [9, 10]. The conflicting results with regard to drug variations may be partially explained by clinical and methodological heterogeneity of these studies. In contrast to our study that focused on antibiotic infusions in infusion bags, previous studies mainly focused on electrolyte solutions or syringe preparations and did not take normal handling into account [4–7, 14, 15]. However, in line with our findings, a study of Layne et al., which examined acetylcysteine infusions, showed no significant difference between the concentrations at the beginning and end of infusions when omitting the mixing step [16]. Also, in the study examining meropenem 1000 mg (reconstituted in 10 mL normal saline) added to 250 mL normal saline showed that mild spontaneous mixing occurred when the mixing step was omitted during preparation, which was attributed to normal handling [8]. Nonetheless, results of different studies are difficult to compare, as the degree of mixing seems to be dependent on many factors, such as the difference in the density of two solutions, viscosity of the drug, the speed of the injection jet, and the needle length [6, 17]. In addition, even the mixing technique may play a role [18, 19]. The extent of spontaneous mixing did not differ between hospital pharmacy and clinical ward preparations, even though the infusions prepared in the hospital pharmacy included an additional transportation route. This suggests that normal handling in the clinical ward seems sufficient to gain a quite homogenous drug concentration. Studies on the influence of normal handling on variations of drug concentrations in infusion containers are scarce. A study of Deardorff et al., examining potassium chloride, suggests that sufficient mixing cannot be assured with normal handling procedures [6]. The different findings may be explained by differences in defining normal handling, physical and chemical properties of investigated drugs, containers used, and in the method of sample collection.

This study has some limitations. First, the concentrations were measured by a method that is standardised for blood and thus deviated from the used matrix. However, there were no differences in internal standards, suggesting reliable measurements. Second, we did not adjust for possible variations in overfill of manufactured infusion bags (generally 5%-10%), which may affect measured concentrations. However, this would not change the interpretation of our results. The strength of our study is that it was conducted in a simulation setting under the standard conditions of the hospital pharmacy and clinical ward environment.

## Conclusions

Our study shows that spontaneous mixing occurs after preparation of the four examined antibiotics (cefuroxime, flucloxacillin, meropenem, and vancomycin), both in hospital pharmacy and clinical ward infusions, when the mixing step after medication addition to the infusion bag is omitted during preparation.

## Data Availability

The data that supports the findings of this study are available from the corresponding author upon reasonable request.

## Abbreviations

I.B.: Ina Barzel
IQR: Interquartile range
m/z: Mass-to-charge ratio
SFC: Super critical fluid chromatography
UPC^2^-MS–MS: Ultra-performance convergence chromatography tandem mass spectrometry
