# Correction to: Evaluation of the importance of mixing during preparation of antibiotic infusions

**DOI:** 10.1186/s40360-022-00566-6

**Published:** 2022-04-28

**Authors:** Ina Barzel, Janique Gabriëlle Jessurun, Soma Bahmany, Paul Hugo Marie van der Kuy, Birgit Catharina Peter Koch, Nicole Geertruida Maria Hunfeld

**Affiliations:** 1 Department of Hospital Pharmacy, Erasmus MC, University Medical Center Rotterdam, P.O. Box 2040, 3000 CA Rotterdam, the Netherlands; 2 Department of Intensive Care, Erasmus MC, University Medical Center Rotterdam, Rotterdam, the Netherlands

**Correction to: BMC Pharmacol Toxicol 23, 22 (2022)**

**https://doi.org/10.1186/s40360-022-00562-w**

Following publication of the original article [1], the authors identified an error in the authors’ list.

The correct authors statement list is that Ina Barzel and Janique G. Jessurun are joint first author.

The original article [1] has been corrected.
